# Region of interest selection in heterogeneous digital image: Wine age prediction by comprehensive two-dimensional gas chromatography

**DOI:** 10.1016/j.crfs.2024.100725

**Published:** 2024-03-29

**Authors:** Nemanja Koljančić, Larissa Onça, Liudmyla Khvalbota, Olga Vyviurska, Adriano A. Gomes, Ivan Špánik

**Affiliations:** a Institute of Analytical Chemistry, Faculty of Chemical and Food Technology, Slovak University of Technology in Bratislava, Radlinského 9, 812 37, Bratislava, Slovakia; b Instituto de Química, Universidade Federal Do Rio Grande Do Sul, Avenida Bento Gonçalves, 9500, 91501-970, Porto Alegre, RS, Brazil

**Keywords:** *Wine analysis*, *Comprehensive two-dimensional gas chromatography*, *Region of interest*, *Volatile organic compounds*, *Wine age*

## Abstract

This study integrates genetic algorithm (GA) with partial least squares regression (PLSR) and various variable selection methods to identify impactful regions of interest (ROI) in heterogeneous 2D chromatogram images for predicting wine age. As wine quality and aroma evolve over time, transitioning from youthful fruitiness to mature, complex flavors, which leads to alterations in the composition of essential aroma-contributing compounds. Chromatograms are segmented into subimages, and the GA-PLSR algorithm optimizes combinations based on grayscale, red-green-blue (RGB), and hue-saturation-value (HSV) histograms. The selected subimage histograms are further refined through interval selection, highlighting the compounds with the most significant influence on wine aging. Experimental validation involving 38 wine samples demonstrates the effectiveness of this approach. Cross-validation reduces the PLS model error from 2.8 to 2.4 years within a 10 × 10 subset, and during prediction, the error decreases from 2.5 to 2.3 years. The study presents a novel approach utilizing the selection of ROI for efficient processing of 2D chromatograms focusing on predicting wine age.

## Highlights

• Digital images are crucial in chemical analysis by detecting color changes.
• GC × GC-TOF-MS is a powerful tool for wine age prediction.
• Selecting the correct ROI is essential for accuracy in heterogeneous images.
• The GA coupled with PLSR enhances image analysis for wine age prediction.

## Graphical abstract

**Figure:**
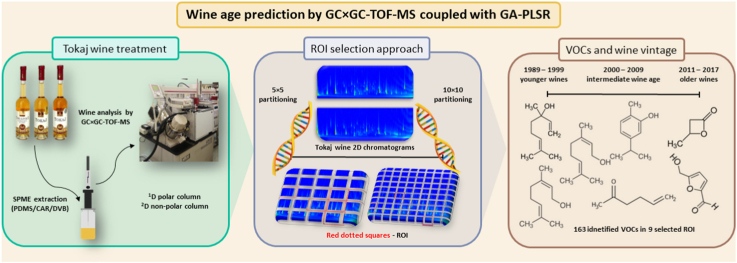

## Nomenclature

2D: two-dimensional
CMYK: cyan, magenta, yellow, key
DI: digital image
GA: genetic algorithm
GC × GC-TOF-MS: comprehensive two-dimensional gas chromatography time-of-flight mass spectrometry
HSV: hue-saturation-value
*i*PLS: interval partial least square
*i*SPA-PLS: interval by successive projection algorithm partial least square
LOOCV: leave one out cross validation
PLSR: partial least square regression
RGB: red-green-blue
RMSECV: root mean square error of cross validation
RMSEP: root mean square error of prediction
ROI: regions of interest
SPME: solid phase microextraction
VOCs: volatile organic compounds

## 1 Introduction

A digital image is a two-dimensional (2D) representation of a real scene captured by a device (including cameras, smartphones and scanners for example), which involves a process of discretization and the use of a mathematical model to represent the color (Capitán-Vallvey et al., 2015). In addition to red-green-blue (RGB) color space, which is the best known, other models are also described in the literature such as CIE XYZ, hue-saturation-value (HSV), CIELAB, cyan, magenta, yellow, key (CMYK) and grayscale color spaces (Byrne et al., 2000). On the other hand, time-of-flight mass spectrometers (TOF-MS) in combination with comprehensive gas chromatography (GC × GC) represents one of the most powerful analytical tools for the separation and identification of unknown compounds in complicated matrices, generating data with a large amount of information. Therefore, work on processing the received data (2D chromatograms) can be demanding and time consuming, and the use of automated software would be beneficial for fast data processing (Stefanuto et al., 2021). With the high availability of low-cost digital image (DI) capture devices in the last few years, the use of digital images as an analytical signal has grown exponentially. This is not surprising considering that many chemical processes involve color changes due to the formation of a product or consumption of a reagent (Khanal et al., 2021). A quick search of the literature shows a multitude of applications of digital imaging as a chemical analysis tool (Meenu et al., 2021). These approaches range from using the color value to obtain an analytical curve (Böck et al., 2022; Gonçalves et al., 2023) to frequency histograms for different color models. This makes it possible to acquire a vector (**x**_1 × J_) of information by samples resulting in a matrix (**X**_I × J_) for a set of *I* samples. Subsequently, the matrices are used as input for multivariate classification (Fernandes et al., 2023) and/or multivariate calibration models (Belén et al., 2020; Vallese et al., 2021). Although less common, there are also DI applications into multiway data acquisition for both calibration and classification (Belén et al., 2020; Vallese et al., 2021). One common point in almost all DI applications is the fact that treated images are completely homogeneous and therefore the definition of a region of interest (ROI) is a trivial task (Meenu et al., 2021). Even when images are captured from heterogeneous samples, this is not addressed as an important factor, while it should be (da Silva et al., 2018).

The basic idea behind the use of DI analytical methodologies is that color values in each pixel are proportional to the concentration of species that produce colors. Therefore, this information can be accessed by multivariate models for the purpose of predicting the concentration of one or more target species or even physical-chemical parameters. In addition, color variations can be used to differentiate groups of samples which allow the use of DI as input to classifiers. Often model improvements are achieved by performing variable selection on the histograms extracted from the ROI. The conventional path to attaining results is reliable when dealing with homogeneous images. However, the same may not be true when dealing with heterogeneous images. We refer to homogeneous and heterogeneous images in the context described França et al., 2017, where spatial distribution of the pixel intensities is the same in the ROI. An image of a solution is considered completely homogeneous, while the image of a solid mother of a solution will have a certain degree of homogeneity, being considered heterogeneous or inhomogeneous.

Interesting approach was developed by Synovec and co-workers and is based on data treatment of 2D chromatograms with supervised tile-based Fisher-ratio analysis across all sample classes (Marney et al., 2013). In this method, the Fisher-ratio is calculated for within a small, rectangular section/region (i.e., tile) of the chromatogram on a per-mass channel (*m*/*z*) basis which significantly shortens the analysis time, the possibility of false positives, as well as an increase in sensitivity, since ideally one tile covers only one peak, which prevents interference with other peaks. The software was successfully used for non-target fingerprinting of metabolome and aroma compounds in environmental, food and beverage samples (Mikaliunaite and Synovec, 2022; Parsons et al., 2015; Schöneich et al., 2022; Sudol et al., 2022; Titaley et al., 2018; Zou et al., 2022). Another method utilized in chromatogram analysis is the pixel-based approach. Both pixel-based and digital image approaches can detect minimal statistical differences between samples and utilize raw data. Pixel-based analyses examine individual pixels, whereas ROI selection focus directly on segments of chromatograms chosen by specific methods. Pixel-based analysis requires meticulous parameter selection for alignment, weighting, and model construction, often necessitating manual adjustments based on visual inspection. Additionally, data preprocessing involves adjusting chromatographic signals based on pixel-by-pixel relative standard deviation (Alexandrino et al., 2019; Freye et al., 2020). Conversely, ROI selection offers automated feature selection through GA, efficiently handles high-dimensional image data, and allows flexibility in parameter tuning for GA and PLSR (Vyviurska et al., 2023). However, computational intensity poses a challenge, especially with large datasets.

The key question that we will address here is, if the image is heterogeneous: are there any regions whose color variability is directly linked to the problem addressed? If the answer to this question is yes, we can consider that the previous selection of ROI with the intention of discarding useless and/or redundant information before the extraction of frequency histograms can promote improved results. Sequentially, the histogram coming from the best ROI can still be subjected to the selection of variables to eliminate color levels that are not directly linked to the analytical problem addressed. The aim of this study is to investigate the integration of the genetic algorithm (GA) with partial least squares regression (PLSR) and various variable selection methods. The goal is to identify the most impactful ROI in heterogeneous images. The proposed strategy is applied in the prediction of wine age by means of 2D chromatogram images obtained by comprehensive chromatography.

## 2 Experimental

### 2.1 Samples and chemicals

All 38 wine samples (vintage from 1989 to 2017) used in this work were obtained directly from Slovak Tokaj wine producers, namely Tokaj & CO, Zlatý Strapec and J&J Ostožovič. The samples include three groups of wines: botrytized wines, varietal wines as well as wine essences. Additional information regarding the samples is given in Table 1. Sodium chloride was obtained from Chemapol (Prague, Czech Republic). A mixture for alkanes (C7–C30), used to calculate retention indices was obtained from Supelco (Belleforte, PA, USA).

**Table 1:** List of wine samples examined in the study, showing origin, category, and vintage.

Sample	Producer	Wine category	Year	aSample set
1	J&J Ostrožovič	Selection essence	2017	C
2	J&J Ostrožovič	Selection essence diluted 2:1	2017	C
3	Tokaj & Co.	Samorodné dry	2015	C
4	Tokaj & Co.	5-Putňa selection	2015	C
5	Tokaj & Co.	Samorodné dry	2011	C
6	Nagy	Zeta	2011	C
7	Tokaj & Co.	Lipovina	2015	C
8	Tokaj & Co.	Muscat yellow	2015	P
9	Tokaj & Co.	Tokaj forditáš	2011	P
10	Tokaj & Co.	4-Putňa selection	2009	P
11	Tokaj & Co.	6-Putňa selection	2006	C
12	Tokaj & Co.	Selection essence	2009	P
13	Tokaj & Co.	6-Putňa selection	2011	C
14	Tokaj & Co.	6-Putňa selection	2011	C
15	Tokaj & Co.	Samorodné dry	2011	C
16	Nagy	Muscat yellow	2013	C
17	Nagy	3-Putňa selection	2006	C
18	Nagy	Lipovina	2013	C
19	Nagy	6-Putňa selection	2009	C
20	Tokaj & Co.	2-Putňa selection	1989	C
21	J&J Ostrožovič	3-Putňa selection	1999	C
22	J&J Ostrožovič	6-Putňa selection	1993	C
23	J&J Ostrožovič	Selection essence	2000	P
24	Zlatý Strapec	5-Putňa selection	1993	P
25	Zlatý Strapec	6-Putňa selection	1993	P
26	Zlatý Strapec	4-Putňa selection	1993	C
27	Zlatý Strapec	3-Putňa selection	1995	C
28	Zlatý Strapec	4-Putňa selection	2000	C
29	Zlatý Strapec	Samorodné dry	1997	C
30	Tokaj & Co.	Samorodné sweet	1997	C
31	Tokaj & Co.	2-Putňa selection	1990	C
32	Tokaj & Co.	6-Putňa selection	1993	P
33	Tokaj & Co.	Selection essence	1999	P
34	Tokaj & Co.	4-Putňa selection	1995	C
35	J&J Ostrožovič	4-Putňa selection	1993	C
36	J&J Ostrožovič	4-Putňa selection	2002	P
37	J&J Ostrožovič	6-Putňa selection	1999	C
38	J&J Ostrožovič	5-Putňa selection	1993	C

a Sample set: C – cross-validation, P – prediction.

### 2.2 Volatile organic compounds (VOCs) extraction and GC × GC-TOF-MS analysis

Volatile organic compounds (VOCs) from wine samples were extracted by solid phase microextraction (SPME) procedure. Five milliliters of wine together with 0.5 g of NaCl were placed in a 20 ml clear glass vial sealed with hole-caps and PTFE/silicone septa and the solution was stirred at 400 rpm. The incubation of stirred sample solutions took 30 min at 60 °C in order to establish equilibrium between liquid and vapor phases. The extraction of VOCs was performed using 50/30 μm thickness PDMS/CAR/DVB SPME fiber (Supelco, Bellefonte, PA, USA) for 30 min at 60 °C. The fiber was conditioned prior use by heating in the needle heater of the autosampler under the conditions recommended by the manufacturer. Desorption was performed in GC injector in splitless mode at 250 °C for 2 min. The analysis was performed on a Pegasus GC × GC-TOF-MS (LECO Corporation, St. Joseph, MI, USA) consisting of an Agilent 7890B gas chromatograph (Agilent Technologies, Palo Alto, CA, USA), TOF-MS (LECO Corporation, St. Joseph, MI, USA). The GC column setup consists of 30 m × 0.25 mm × 0.25 μm DB-FFAP column (Agilent Technologies, Palo Alto, CA, USA) in the first and 1.39 m × 0.1 mm × 0.1 μm BPX-50 (SGE Analytical Science, Melbourne, Australia) in the second dimension. Helium with purity 99.999% was used as a carrier gas with a flow rate of 1 mL/min. The primary oven program was: 40 °C (15 min) to 220 °C (5 min) at 2 °C/min. Modulator was kept at 15 °C higher temperature compared to actual oven temperature with a modulation period of 10 s. A temperature offset of 5 °C was used for the temperature program in the second dimension. MS ion source temperature was set to 250 °C, mass spectra were obtained at 70 eV ionization energy and 1800 V detector voltage. The signal acquisition rate was 100 spectra/s in the *m*/*z* range 29–550. Three replicates were performed for each sample in sequence. The possible identities of the VOCs present in the wine samples were determined using LECO ChromaTOF 4.51 software, based on comparison with the NIST17 and FFNSC2 mass spectral libraries, utilizing linear retention indices, and with a similarity score greater than 800 (with 1000 being the optimal value). The resulting retention index values were subsequently compared to the reference values obtained from the NIST WebBook Chemistry database.

### 2.3 ROI selection - proposed approach

**Fig. 1:**
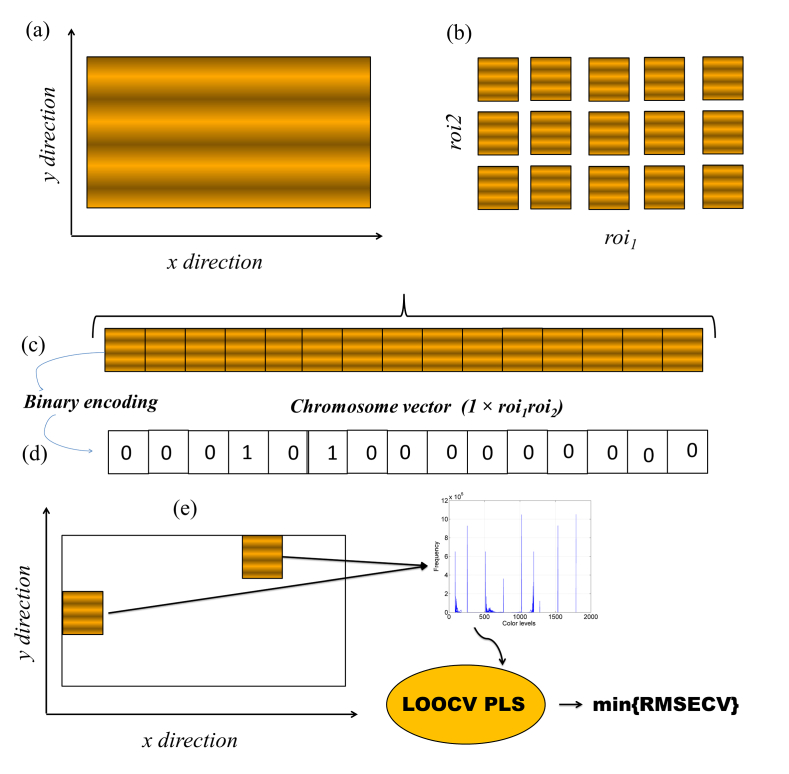
Schematic diagram of the operation of the proposed method: (a) representation of the image to be treated and (b) after being divided into ROI in the x to y directions. In (c) the unfolding process of the image in vector (d) followed by binary coding for GA input is displayed. Illustration of subimages selected in a chromosome extraction of the histograms that are used as input for the PLS LOOCV regression models.

In this proposal an image as depicted in Fig. 1a sized (J × K) is subdivided into *n* subimages (j' × k'). The number of subimages *n* corresponds to the parameters *roi*_*1*_ × *roi*_*2*_ (Fig. 1b), which is user defined and split the image into *roi*_*1*_ columns and *roi*_*2*_ rows. In the sequence, the two-dimensional structure of the image is unfolded (Fig. 1c) in vector (called **r**) whose length will be given by 1 × *roi*_*1*_*roi*_*2.*_ In each **r** position is stored a subimages which is a candidate to be selected by GA coupled to PLSR. The initial step in the GA involves generating a random population and storing it in a matrix referred to as **P**. (sized p × *roi*_*1*_*roi*_*2*_). Here, *p* represents the number of individuals or chromosomes, each containing *roi*_*1*_*roi*_*2*_ genes, constituting the population that will undergo evolution for *g* generations. Each chromosome contains from 1 to *Nim* subimages. The *Nim* parameter is defined by the user, with the limit value *roi*_*1*_*roi*_*2*_ (which corresponds to the entire image). Both *p* and *g* are parameters that must be chosen by the user. The matrix **P** contains the binary coded chromosomes (0 or 1). Where 0 is indicated in the exclusion and 1 in the inclusion of a specific subimage in the PLSR model.

Histograms for all subimage indicated on a specific chromosome are obtained considering grayscale, RGB and HSV or some of those color models arbitrarily chosen by the user. The PLS leave one out cross validation (LOOCV) model is run and the square root of the mean error is stored as an indication of the quality of the respective chromosome to generate good results. The initial population will evolve through an iterative cycle of *g* generations. Between one generation and another the following genetic operators are applied: elitism (10%), mutation (1%) and crossover (60%) via the roulette wheel method with a single division in each chromosome. For a complete description of genetic algorithms applied to variable selection in chemical data see the references (Cho and Hermsmeier, 2002; Leardi and Gonzalez, 1998).

As GA belongs to the group of combinatorial optimization methods known as metaheuristics, its results depend on the starting conditions and at each algorithm run. In order to evaluate the reliability of the selected subimage optional GA could be executed in a Monte Carlo (Allegrini and Olivieri, 2011; Konovalov et al., 2008) inner loop, while different results are obtained. Furthermore, the final histogram derived from the optimal subimages allows for interval selection through two distinct options: intervals PLS (*i*PLS) (Norgaard et al., 2000) and interval by successive projection algorithm PLS (*i*SPA-PLS) (de Araújo Gomes et al., 2013).

### 2.4 Region of interest (ROI) selection

All calculations were performed in MatLab (2012a) environment. GC × GC chromatograms were stored as images in.bmp format and 24 bits, sized 705 height × 1195 width pixels corresponding to the second and first chromatographic dimension respectively. ROI selection based on GA-PLS was implemented in command line format in MatLab. The process of ROI selection was conducted utilizing a genetic algorithm with the following parameters: an initial population of 100 individuals, 100 generations, and mutation and reproduction probabilities set at 5% and 65%, respectively. The set of samples was divided into calibration (28 samples) and prediction (10 samples) via SPXY, samples differences in both instrumental responses (**X** matrix) and wine age (**y** vector) spaces, according to Euclidean distance in order to ensure maximum representativeness in calibration and at same time avoid extrapolation in prediction step (Galvao et al., 2005). Coupled to GA, PLS model was performed with an inner loop for LOOCV (Baumann, 2003) considering the colors RGB frequency histogram extracted from the full image as well as on the RGB histogram of the subset of images selected by the GA.

## 3 Results

### 3.1 Comparative analysis of raw GC × GC chromatograms

**Fig. 2:**
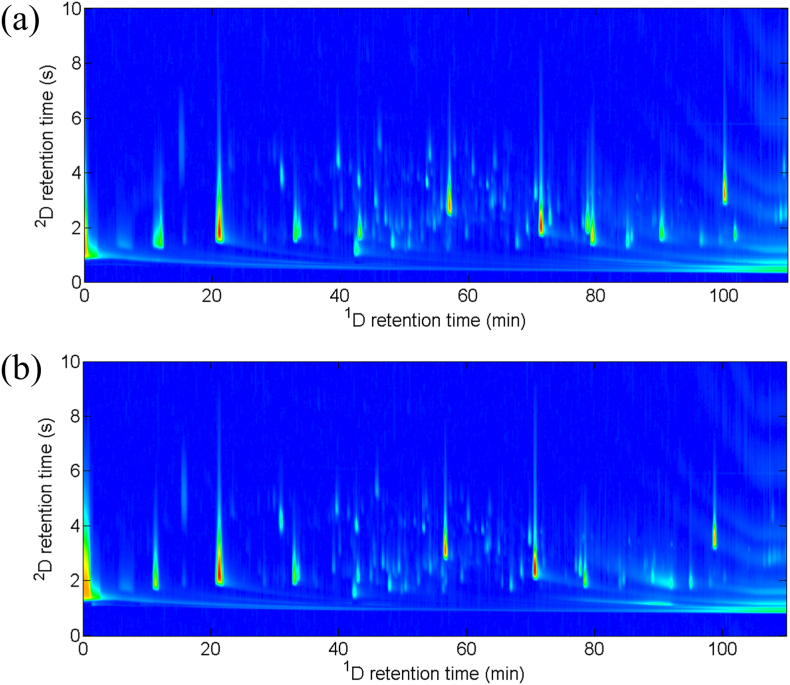
Representative GC × GC-TOF-MS chromatogram of wine samples included in this study. The profiles are displayed for (a) botrytized wine sample and (b) varietal wine sample.

Tokaj varietal and botrytized wines (Fig. 2) are very complex samples. Their VOCs fraction consists of hundreds of compounds, belonging to different chemical classes and the concentration of these compounds vary between ng/L to hundreds mg/L. A rough comparison of the chromatographic profiles of organic compounds was conducted in botrytized and varietal wines across different vintage periods in range from 1989 to 2017. Minor exceptions aside, the results indicate little or no discernible difference between the two types of wine. Botrytized wines were distinguished by a putňa number (the equivalent of the Hungarian "putonny"), indicating the number of barrels containing botrytized grapes added to 130–136 L of fermented wine. However, correlation analysis failed to identify any significant differences between the chromatographic profiles of botrytized wines with different putňa numbers, except for a weaker peak intensities in the botrytized wine sample from 2015. In addition, the comparison of chromatograms of botrytized wine samples with varietal wines resulted in almost identical distribution of organic compounds, including terpenes, esters, ketones, aldehydes, and polyaromatic compounds. The most noticeable variation in the organic compound profiles was found in the wine essence sample. The wine essence sample from the year 2000 exhibited more intense peaks characteristic of esters, as well as a greater presence of naphthalene derivatives. The potential disparity in chromatograms between varietal and botrytized wines lies in the possible presence of low or trace concentrations of certain compounds, whose identification is facilitated by selecting specific ROIs. These compounds similarly contribute to the distinctive aroma of wines produced in the Tokaj wine region. Table 1S presents the retention frames of selected ROI defined by the GA method, which show the highest statistical significance for classifying samples according to vintage. Markers in a specific region, which show potential for differentiating samples according to vintage, belong to different groups of volatile organic compounds. Due to the full chromatograms being complex data sets, this approach enables a more accurate comparison of the profiles of organic compounds in different wine samples.

### 3.2 ROI -GA –PLS in wine age prediction

**Fig. 3:**
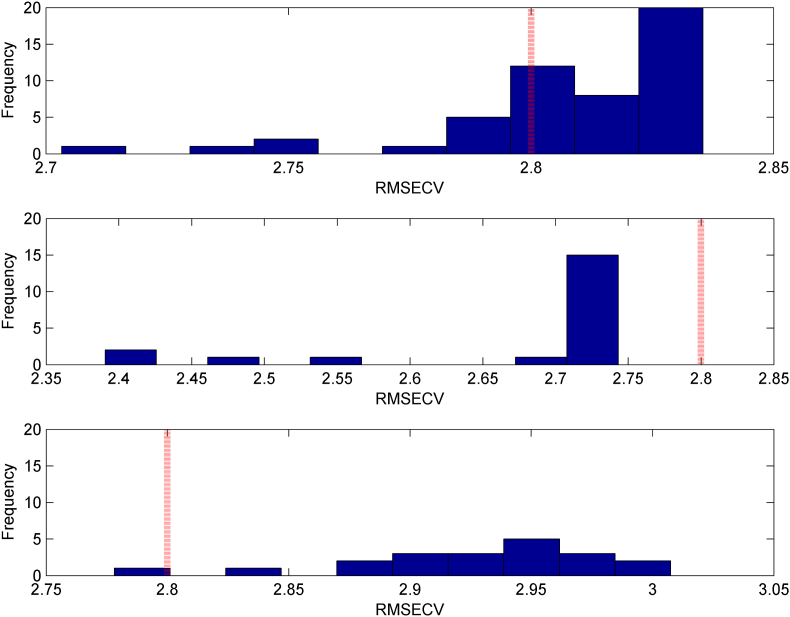
Distribution histogram of the best RMSECV values in each GA generation: images divided into respectively (a) 5 × 5, (b) 10 × 10 and (c) 15 × 15. The red vertical line corresponds to the RMSECV achieved with the full image.

Originally sized images 705 height × 1195 width pixels (Fig. 2) were split into 5 × 5, 10 × 10 and 15 × 15 ROI and subjected to GA. The purpose of this was to select the optimal ROI, with the aim of not just improving accuracy, but also identifying chemical compounds that are linked with the age of wine using a 2D GC × GC image. Fig. 3 depicts the distribution histogram of the best root mean square error of cross validation (RMSECV) values in each GA generation: images divided into (a) 5 × 5, (b) (10 × 10 and (c) 15 × 15. The red vertical dotted line corresponds to the RMSECV achieved with the full image. As can be seen, the best scenario was observed when images were partitioned into 10 × 10 sub-ROI (Fig. 3b).

**Fig. 4:**
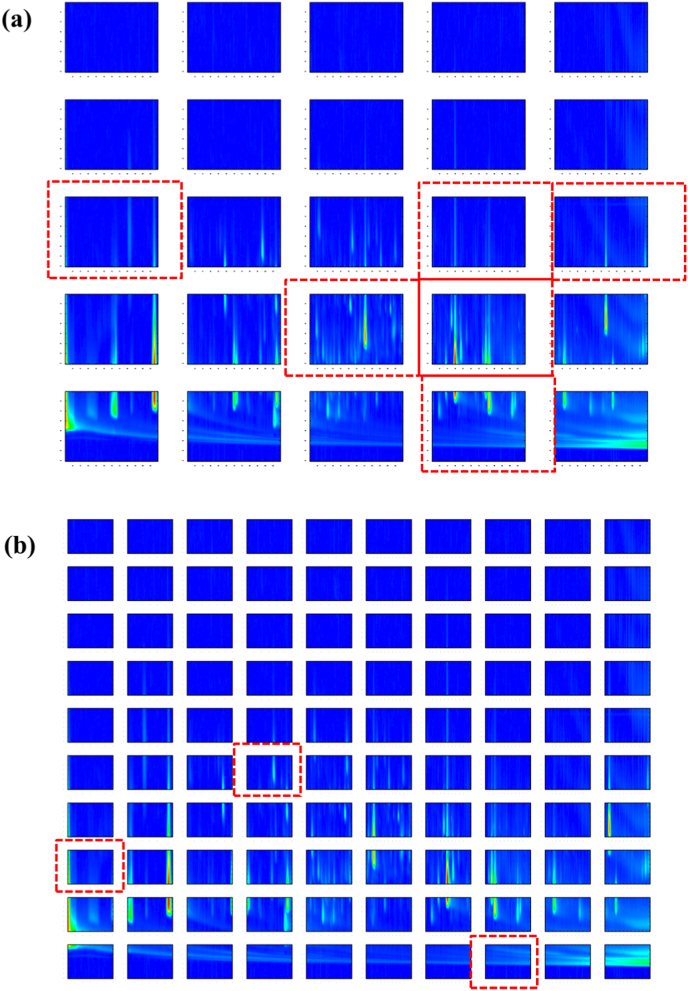
2D image chromatogram partitioned in (a) 5 × 5 and (b) 10 × 10 respectively. The red dotted squares indicate the selected ROI. The x axis represents the retention time in the first dimension (minute); y axis represents the retention time in the second dimension (s).

When the images were partitioned at 5 × 5, most of the RMSECV values were higher than that obtained when the PLS models were based on the full image. On the contrary, 15 × 15 images significantly worsen results in terms of RMSECV. Under GA described condition, optimization was carried out on participation of images 5 × 5 and 10 × 10. The adopted procedure runs the GA 10 times, and the final result is chosen as the best among the 10 attempts. In Fig. 4 shows the selected ROI in both 5 × 5 and 10 × 10 respectively. The selected regions (Table 1S) in both cases show a convergence between the selected regions, but when the images were partitioned 10 × 10, a smaller amount of information was selected. This approach allows identifying the importance of which compounds contributed to the purpose of predicting the wine age.

### 3.3 Comparative analysis of VOCs profile in ROI related to wine age

Table 2S presents the list of compounds identified in specific ROI for the test and cross-validation samples, respectively. Samples were grouped in three distinct groups based on their vintage: older (vintage 1989–1999), intermediate vintage (2000–2009) and younger wines (2011–2017). Out of the selected 10 × 10 matrix regions, mostly peaks belonging to the solvent or siloxanes were identified. Furthermore, a more detailed analysis of the 5 × 5 matrix led to the selection of six statistically significant ROI, with most compounds being identified at positions 2,3 (^1^D 2.0028–3.9915 s, ^2^D 50.0335–69.9665 min), 2,4 (^1^D 2.0028–3.9915 s, ^2^D 70.0503–89.9832 min), and 1,4 (^1^D 0.0000–1.9886 s, ^2^D 70.0503–89.9832 min) (see Table 1, Table 2S). Notably, the compounds found in the regions responsible for discriminating wines based on their age mainly belong to esters, terpens and alcohols. Chemical transformations that occur throughout the process of wine aging result in the modification of concentrations of key aroma-contributing compounds. These compounds encompass a range of VOCs including limonene, linalool, isopentyl hexanoate, nerol, and 2,4-heptadienal (Deibler and Delwiche, 2003). Evidence indicates that these compounds significantly influence the contrast between younger and older wines concerning their presence. These compounds were exclusively identified within the cluster of younger wines, with limonene and linalool also being recognized within the intermediate-aged wine group, while menthol was detected in a single sample of older wines. In addition to the aforementioned compounds, only in the cluster of younger wines esters such as ethyl-11-hexadecenoate, ethyl coumarate, methyl 9-12-octadecadienoate I, methyl-icosa-11,14-dienoate, methyl 9-,12-octadecadienoate II, methyl decanoate, ethyl dodecanoate, ethyl pentadecanoate, methyl hexadecanoate, methyl benzoate, ethyl 9-decenoate, ethyl 4-acetoxybutanoate, benzyl acetate, and 2-phenylethyl acetate were identified. This is consistent with the observations that oxidative processes contribute to the reduction in specific ester levels (Wang et al., 2019; Yu et al., 2022). Furthermore, the presence of specific furan derivatives, such as methyl-2-furancarboxaldehyde, has been revealed to play a role in distinguishing wines based on their age. These compounds are predominantly found in older wines, adding to their distinctive characteristics (Furdíková et al., 2019, 2020; Wang et al., 2019). Furan and its derivatives are thought to be formed by carbohydrate dehydration followed by cyclization in Maillard-type reactions and are believed to accumulate during later stages of wine aging, as well as being introduced from wood during wine aging (Wang et al., 2020). During the aging process of wine, a notable phenomenon connected to acid esterification occurs. This leads to an increase in the concentration of certain esters such as 3-methylbutyl-2-hydroxypropanoate. Simultaneously, there is a decline in the concentration of lower alcohols such as glycol derivatives, 1-decanol, 1-dodecanol, 4-decen-1-ol, 3-hepten-2-ol, and 2-phenylethanol (Guillén et al., 2005; Wang et al., 2020; Yu et al., 2022). Apart from butyrolactone and 1-phenyl-1,2-propanedione, which exhibit higher concentrations in older wines, carvacrol exhibits greater prevalence in intermediate and younger wines. On the contrary, the content of lactones and ketones did not influence the distribution of wines based on their age. This is due to their nearly uniform distribution across all samples. The presence of ketone compounds can arise through two distinct mechanisms: either via the oxidation of fatty acids and their corresponding alcohols, or through the degradation of amino acids and sugars (Aznar et al., 2003; Culleré et al., 2004; Yu et al., 2022). Phenolic compounds, including (dimethylethyl)phenol and certain ethyl-phenols, undergo a concentration increase within wine ages. This is attributed to their extraction from the wine barrels, a process inherent to the aging progression (Wang et al., 2019; Yu et al., 2022). While naphthalene derivatives are not typically considered to be major contributors to wine quality and aging, their behavior are depended also on other factors, such as the type of wine, the wine storage conditions, and the presence of other chemical compounds (Chatonnet and Escobessa, 2007). The outcomes of this study point to a generally similar content of naphthalene across all the samples data. The results obtained align with previous research findings (Furdíková et al., 2019; Furdíková et al., 2020; Khvalbota et al., 2021; Machyňáková et al., 2021; Vyviurska et 2021; Vyviurska et al., 2022). Compounds such as ethyl octanoate, benzaldehyde, butyl ethyl succinate, β-phenethyl acetate, and sorbic acid were detected in all samples (Table 2S). Histograms extracted (Fig. 1S) from the full image and selected regions were used to obtain the final PLS models and the statistical summary of cross validation is displayed in Table 2.

**Table 2:** Statistical summary of cross validation and prediction.

Models	^a^LV	^b^RMSECV (years)	^c^REP_cv_ (%)	^d^R^2^_cv_	^e^RMSEP (years)	^f^REP (%)	^g^R^2^	Bias**
Full image	2	2.8	0.14	0.95	2.5	0.12	0.95	absent (t_cal_ = 1.0938)
GA (5 × 5)	2	2.8	0.14	0.95	2.3	0.11	0.96	absent (_tcal_ = 1.0938)
GA (10 × 10)	3	2.4	0.11	0.96	2.3	0.11	0.96	absent (t_cal_ = 0.8782)

^a^LV – latent variables; **(t_crit_ = 1.8331 at 95%); ^b^RMSECV – root mean square error of cross validation; ^c^REP_CV_ – reproducibility of cross-validation; ^d^R^2^_CV_ – coefficient of determination of cross validation; ^e^RMSEP – root mean square error of prediction; ^f^REP – reproducibility; ^d^R^2^ – coefficient of determination.

It can be noticed that although if the images were partitioned in 5 × 5 there was no improvement in the accuracy of the results. When the images were divided into 10 × 10 it was possible to notice an improvement in the figures of merit. But the most significant contribution is the possibility of finding only compounds connected with the parameter of interest, in this case the age of the wine. In Fig. 2S the regression coefficients of the model based on the full image are shown; note that none infers about the contribution of the chemical composition on the age of the wine from them.

As a final step to assess predictive ability, the models were employed to predict the age of wine samples in an independent sample set. The statistical summary of prediction is shown in Table 2. When applied to the sample set that did not participate in the modeling phase both approaches based on selection ROI show similar and slightly better results than models based on full image. For all cases on 95% confidence level, no significant bias was observed.

## 4 Conclusion

The study highlights the importance of considering color variability in heterogeneous images and proposes a method to identify the regions whose color variability is directly linked to the analytical problem. The proposed approach involves dividing the chromatogram into subimages and creating a random initial population of chromosomes using binary coding. The GA coupled with PLSR evaluates the quality of each chromosome based on histograms obtained from the selected subimages. The population undergoes generational evolution through the application of genetic operators such as elitism, mutation, and crossover. The goal was to identify the best ROI or combination of subimages that provide accurate predictions of wine age. Experimental validation was performed using 38 wine samples from different producers and categories. The volatile organic compounds in the wine samples were extracted using SPME and analyzed using GC × GC-TOF-MS technique. The obtained GC × GC chromatograms were used for ROI selection. Following the analysis, distinct compounds were identified within the previously established region of interest (ROI), and these compounds are linked to the age of the wine. These compounds predominantly include esters, alcohols, and terpenes. This research provides a novel strategy for ROI selection in heterogeneous images and demonstrates its application in the prediction of wine age using GC × GC-TOF-MS data. It has been demonstrated that slightly better results were obtained when predicting the age of the wine using ROI with a significant 95% confidence level. The approach has the potential to be applied in other analytical fields where heterogeneous images are encountered and accurate predictions or classifications are required.

## CRediT authorship contribution statement

**Nemanja Koljančić:** Data interpretation, Methodology, Writing – original draft, Review & Editing. **Larissa Onça:** Data interpretation, Methodology, Writing – original draft, Review & Editing. **Liudmyla Khvalbota:** Investigation, Methodology, Writing – original draft. **Olga Vyviurska:** Investigation, Data interpretation, Methodology, Writing – original draft, Review & Editing. **Adriano A. Gomes:** Data interpretation, Methodology, Writing – original draft, Review & Editing. **Ivan Špánik:** Conceptualization, Writing – original draft, Review & Editing, Funding acquisition, Supervision, Project administration.

## Declaration of competing interest

The authors declare that they have no known competing financial interests or personal relationships that could have appeared to influence the work reported in this paper.

## Data Availability

Data will be made available on request.
