# Supplementary material for: Region of interest selection in heterogeneous digital image: Wine age prediction by comprehensive two-dimensional gas chromatography
Source: Curr Res Food Sci. 2024 Mar 29;8:100725. doi: 10.1016/j.crfs.2024.100725 (PMC11000173; doi:10.1016/j.crfs.2024.100725)
Supplement: Multimedia component 1 [file mmc1.docx]

**Supplementary Material**

**Region of interest selection in heterogeneous digital image: Wine age prediction by comprehensive two-dimensional gas chromatography**

Nemanja Koljančić^a^, Larissa Onça^a,b^, Liudmyla Khvalbota^a^, Olga Vyviurska^a^, Adriano A. Gomes^a,b^ and Ivan Špánik^a*^

*^a^Institute of Analytical Chemistry, Faculty of Chemical and Food Technology, Slovak University of Technology in Bratislava, Radlinského 9, 812 37 Bratislava, Slovakia*

*^b^Instituto de Química, Universidade Federal do Rio Grande do Sul, Avenida Bento Gonçalves, 9500, 91501-970, Porto Alegre, RS, Brazil*

**_______________________________**

Corresponding author

*e-mail: [ivan.spanik@stuba.sk](mailto:ivan.spanik@stuba.sk) Phone: +421 2 59325277


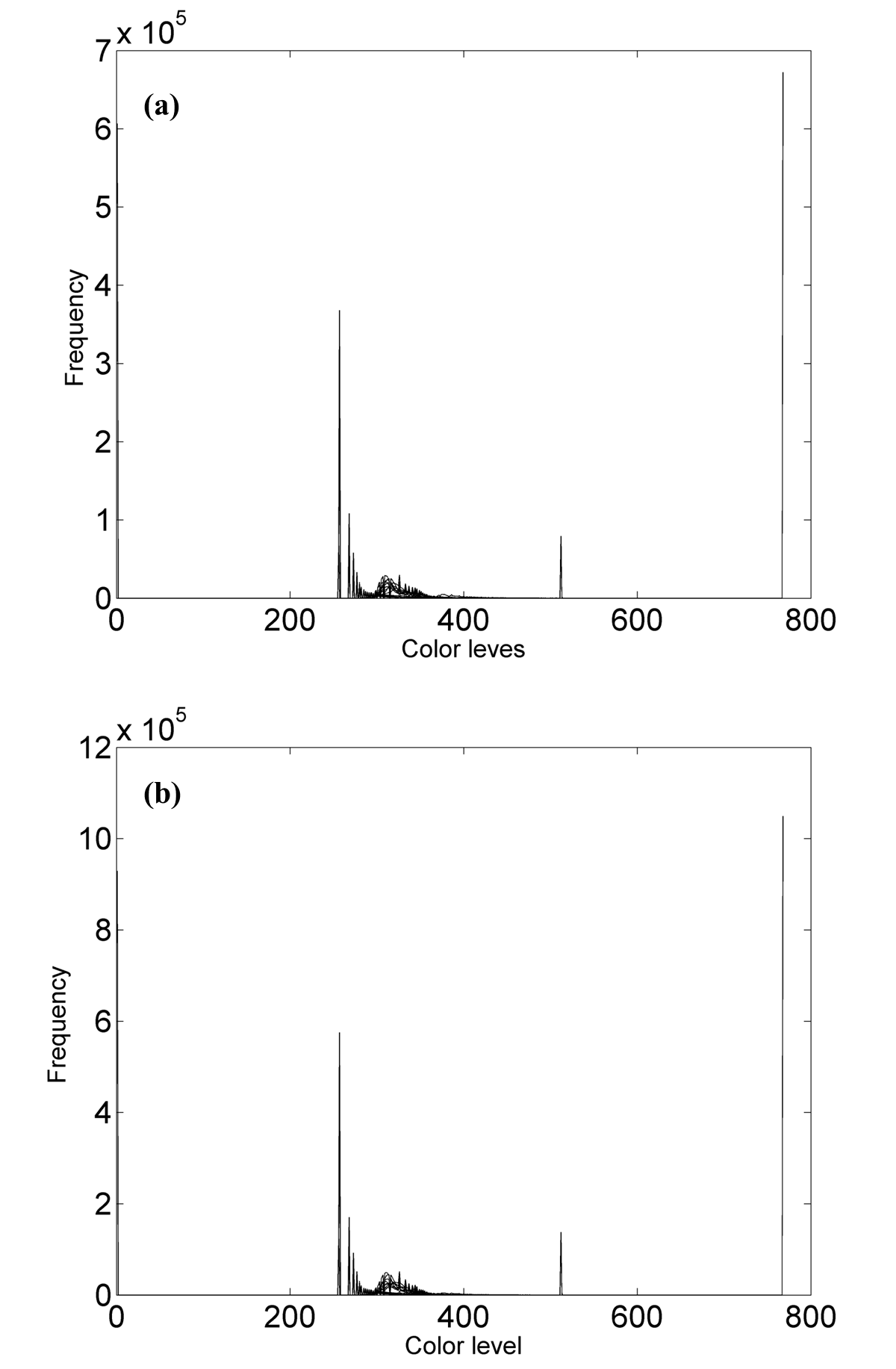


**Figure 1S**: RGB color histograms in (a) the full image and (b) the regions selected for 10 x 10 paritioning.

**
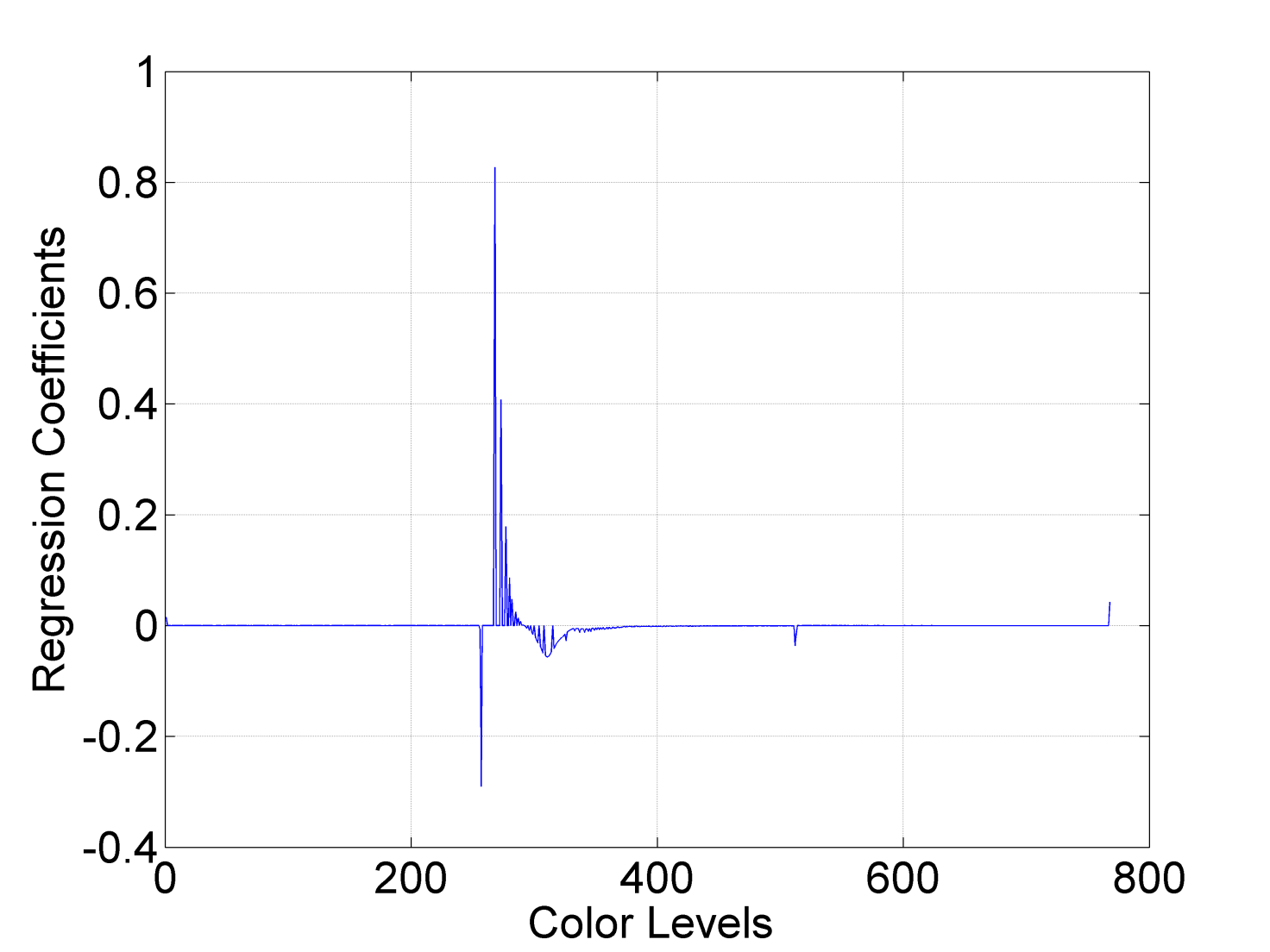
**

**Figure 2S**: Regression coefficients of the model based on the full image

**Table 1S**: Retention times of the selected regions.

|  |  | **^2^D (sec)** | | **^1^D (min)** | |
| --- | --- | --- | --- | --- | --- |
| **10 × 10 ROI** | ^a^**Postion** | **Start** | **Finish** | **Start** | **Finish** |
| ROI1 | 3,1 | 2.0170 | 3.0114 | 10.0000 | 19.9665 |
| ROI2 | 5,4 | 4.0341 | 5.0284 | 40.1508 | 50.1173 |
| ROI3 | 1,8 | 0.0000 | 0.9943 | 80.1843 | 90.0670 |
|  |  | **^2^D (sec)** | | **^1^D (min)** | |
| **5 × 5 ROI** | ^a^**Postion** | **Start** | **Finish** | **Start** | **Finish** |
| ROI4 | 3,1 | 4.0057 | 5.9943 | 10.0000 | 29.9330 |
| ROI5 | 2,3 | 2.0028 | 3.9915 | 50.0335 | 69.9665 |
| ROI6 | 3,4 | 4.0057 | 5.9943 | 70.0503 | 89.9832 |
| ROI7 | 2,4 | 2.0028 | 3.9915 | 70.0503 | 89.9832 |
| ROI8 | 1,4 | 0.0000 | 1.9886 | 70.0503 | 89.9832 |
| ROI9 | 3,5 | 4.0057 | 5.9943 | 90.0670 | 110.0000 |

^a^Column and row of the selected ROI in Fig. 4
